# The searching for autism biomarkers: a commentary on: a new methodology of viewing extra-axial fluid and cortical abnormalities in children with autism via transcranial ultrasonography

**DOI:** 10.3389/fnhum.2014.00240

**Published:** 2014-05-12

**Authors:** Dario Siniscalco

**Affiliations:** ^1^Department of Experimental Medicine, Second University of NaplesNapoli, Italy; ^2^Centre for Autism - La Forza del SilenzioCaserta, Italy; ^3^CancellautismoFlorence, Italy

**Keywords:** autism, transcranial ultrasonography, biomarkers, brain lesions and behavior disturbances, neurotransmitter agents

Autism spectrum disorders (ASDs) are now recognized as urgent public health concerns with dramatically increased frequencies seen across Western countries for more than 2 decades (Baio, 2014). ASDs significantly impact on the quality of life for both patients and their families (Siniscalco et al., 2013). It has been estimated that the total lifetime societal cost of caring for one individual with ASDs is about $3.2 million US dollars (Siniscalco, 2013).

The diagnosis of ASDs is based on the evaluation of social communication skills and behavior; currently there is no specific biomarker rendering a diagnosis potentially subjective. Recent changes with the DSM-5 diagnostic criteria illustrate the challenges clinicians face due to the lack of quantifiable diagnostic markers. The search for biomarkers is a priority in autism research and care (Siniscalco et al., 2012).

The authors Bradstreet et al. (2014) used a novel imaging approach to detect abnormalities in the central nervous system of autistic subjects. The authors used a transcranial ultrasonography (TUS) imaging technique, that allowed the detection of abnormalities in extra-axial fluid production and cortical structures. Alternative brain imaging technologies such as MRI are generally expensive and invasive, especially for children with ASDs who need sedation to undergo such investigation. TUS resolution is potentially capable of observing structures as small as 0.1 mm and can detect a cortical abnormality up to a depth of several cm in order of magnitude. Using this approach the authors were able to identify a cortical dysplasia and increased extra-axial fluid presence in brains of autistic patients. These brain abnormalities correlated with autism severity. In addition, this study raised the possibility that the detected cortical abnormalities may be associated with impaired neurotransmissions which could underlie some of the cognitive abnormalities seen in ASDs.

This very interesting technique is simple, reproducible, safe, and effective. It is non-invasive and presents the advantage of being inexpensive. It further has the potentials to enable real-time analysis of sub-arachnoidal and cortical brain regions. Functional imaging studies conducted in autism have shown consistently abnormalities of hypo-perfusion in the temporal lobe areas which may correlate with the degree of impairment. Unfortunately most ASD subjects do not have access to such analysis, due to limited access and cost. Clinicians are in need of fast, non-invasive, accurate and safe means to evaluate any potential structural brain abnormality, especially if such abnormalities correlate with mental and neurological dysfunctions. 2D TUS might offer such fast analysis of the upper brain anatomy. Whether this can assist with regard to diagnosis remains to be shown with larger scale investigations and comparison with age matched individuals suffering from other developmental abnormalities.

## Conflict of interest statement

The author declares that the research was conducted in the absence of any commercial or financial relationships that could be construed as a potential conflict of interest. The reviewer Dr. Sapone declares that, despite having collaborated with the authors, the review process was handled objectively.
